# MethylExtract: High-Quality methylation maps and SNV calling from whole genome bisulfite sequencing data

**DOI:** 10.12688/f1000research.2-217.v2

**Published:** 2014-02-21

**Authors:** Guillermo Barturen, Antonio Rueda, José L. Oliver, Michael Hackenberg

**Affiliations:** 1 Dpto. de Genética, Facultad de Ciencias, Universidad de Granada, Granada, 18071, Spain; 2 Lab. de Bioinformática, Inst. de Biotecnología, Centro de Investigación Biomédica, Granada, 18016, Spain

## Abstract

Whole genome methylation profiling at a single cytosine resolution is now feasible due to the advent of high-throughput sequencing techniques together with bisulfite treatment of the DNA. To obtain the methylation value of each individual cytosine, the bisulfite-treated sequence reads are first aligned to a reference genome, and then the profiling of the methylation levels is done from the alignments. A huge effort has been made to quickly and correctly align the reads and many different algorithms and programs to do this have been created. However, the second step is just as crucial and non-trivial, but much less attention has been paid to the final inference of the methylation states. Important error sources do exist, such as sequencing errors, bisulfite failure, clonal reads, and single nucleotide variants.

We developed *MethylExtract*, a user friendly tool to: i) generate high quality, whole genome methylation maps and ii) detect sequence variation within the same sample preparation. The program is implemented into a single script and takes into account all major error sources. *MethylExtract* detects variation (SNVs – Single Nucleotide Variants) in a similar way to *VarScan*, a very sensitive method extensively used in SNV and genotype calling based on non-bisulfite-treated reads. The usefulness of *MethylExtract* is shown by means of extensive benchmarking based on artificial bisulfite-treated reads and a comparison to a recently published method, called *Bis-SNP*.

*MethylExtract* is able to detect SNVs within High-Throughput Sequencing experiments of bisulfite treated DNA at the same time as it generates high quality methylation maps. This simultaneous detection of DNA methylation and sequence variation is crucial for many downstream analyses, for example when deciphering the impact of SNVs on differential methylation. An exclusive feature of *MethylExtract*, in comparison with existing software, is the possibility to assess the bisulfite failure in a statistical way. The source code, tutorial and artificial bisulfite datasets are available at http://bioinfo2.ugr.es/MethylExtract/ and http://sourceforge.net/projects/methylextract/, and also permanently accessible from 10.5281/zenodo.7144.

## Introduction

DNA methylation at the cytosine carbon 5 position (5meC) is an important epigenetic mark in eukaryotic cells that is predominantly found in CpG or CpHpG (H = A,C,T) sequence contexts ^1^. Epigenetic modifications at the DNA level play important roles in embryonic development ^2, 3^, transcription ^4^, chromosome stability ^5^, genomic imprinting ^6^ and in the silencing of transposons in plants ^7^. Furthermore, aberrant methylation is involved in the appearance of several disorders as cancer, immunodeficiency or centromere instability ^8^. The methylation pattern along the genome sequence carries biologically relevant information. For example: methylated promoter regions are generally associated with silenced transcription and DNA methylation in the gene body of transcribed genes is often increased ^8^. Given these findings, the generation of high quality whole genome methylation maps at a single cytosine resolution is an important step towards the understanding of how DNA methylation is involved in the regulation of gene expression or the generation of a pathologic phenotype. In addition, methylation maps may provide new insights into how the methylation patterns themselves are established.

**Figure 1.**
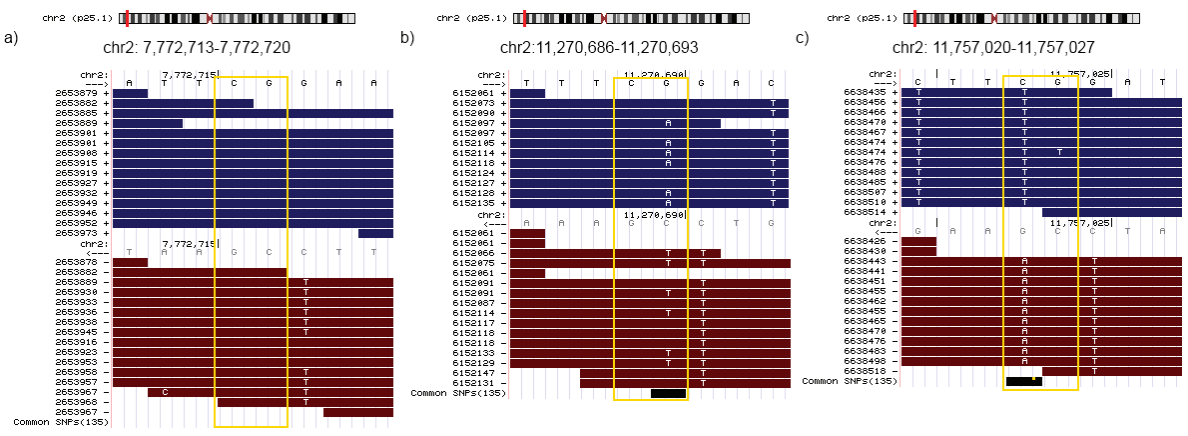
SNV detection in bisulfite converted reads. Sequence variation can be detected for a cytosine position analyzing the nucleotide frequency at the same position but on the complementary strand. Bisulfite conversion does not affect the guanine on the complementary strand, therefore the presence of any other base (H=A,C,T) might indicate the existence of an SNV. The figure illustrates three different situations: ( **a**) a methylated cytosine in a CpG context without sequence variation (all reads that map to the position independently of the strand carry a cytosine in the corresponding position), ( **b**) a heterozygous SNV (genotype C/T, SNV detected on the ‘+’ strand) and ( **c**) a homozygous SNV (genotype T/T, SNV detected on the ‘-’ strand). The example in **b**) shows a heterozygous SNV; the 6 reads with A/G mismatch from a total of 11 reads mapping the position indicate a heterozygous variation. Furthermore, we can conclude that the cytosine allele is methylated (7 reads with C/C matches to the ‘-’ strand). The case illustrated in part **c**), shows 12 reads that show C/T mismatch (‘+’ strand in blue in the upper part). Without looking at the complementary strand, the inference would be a completely un-methylated cytosine. However, the 11 reads that map to the complementary strand show an A/G mismatch at the corresponding position (we would expect guanines in the case of bisulfite conversion). Note that on bisulfite treated datasets only G/A mapped on the ‘+’ strand and C/T on the ‘-’ strand (refereed to the ‘+’ strand) can be used for SNV calling purposes. The figure was generated using the UCSC Genome Browser ^41^.

Several high-throughput techniques have been developed able to generate whole genome methylation maps. In general, the techniques consist of a methylation-sensitive pre-treatment and a read-out step. The pre-treatments generally consist of digestion by methyl-sensitive endonucleases, methyl-sensitive immunoprecipitation or bisulfite conversion, while the read-out of the methylation information is done by hybridization, amplification or sequencing ^9^. Recently, several promising techniques have been developed that link the bisulfite conversion with High-Throughput Sequencing (MethylC-Seq ^10^, BS-Seq ^11^ or RRBS ^12^). Briefly, the bisulfite treatment converts un-methylated cytosines into uracil (converted to thymine after PCR amplification) while leaving methylcytosines unconverted. After sequencing the bisulfite-treated genomic DNA, the methylation state can be recovered from the sequence alignments. Therefore, the methylation profiling from High-Throughput Bisulfite Sequencing data can be divided into two steps: the alignment of the reads, and the read-out of the methylation levels from the alignment. The alignment of bisulfite-treated reads is highly non-trivial due to the reduced sequence complexity given that all cytosines except methylcytosines are converted to thymines. This challenge has been extensively addressed over the last years and several algorithms are available that either align the reads in a 3-letter space or adapt the alignment scoring matrix in order to account for the C/T conversions. Among these algorithms are *BSMAP* ^13^, *Bismark* ^14^, *MethylCoder* ^15^, *NGSmethPipe* ^16^, *BS Seeker* ^17^, *Last* ^18^ and *BRAT-BW* ^19^. Note that some of these tools are not just alignment programs but can, in addition, perform the profiling of the methylation levels such as *Bismark* and *MethylCoder*. After alignment, the methylation states can be recovered: C/T mismatches indicate un-methylated cytosines while C/C matches reveal methylcytosines. However, several error sources−like sequencing errors, clonal reads, sequence variation, bisulfite failure and mis-alignments−can lead to a wrong inference of the methylation levels ^16, 18, 20^. For example, C→T or T→C (on converted cytosines) sequencing errors would be incorrectly interpreted as un-methylated or methylated respectively biasing the results towards lower or higher methylation levels. On the other side, bisulfite failures bias the methylation levels only to higher levels; un-methylated cytosines are not converted and therefore detected as methylcytosines. The existence of sequence variation is another important error source that was traditionally disregarded in the data analysis of whole genome bisulfite sequencing (WGBS) experiments. A C/T SNV would be interpreted as un-methylated cytosine. Given that over two thirds of all Single Nucleotide Polymorphisms (SNPs) occur in a CpG context, having two alleles: C/T or G/A ^21^, sequence variation needs to be addressed as an important error source. A C/T SNV manifests on the complementary DNA strand as an adenine, while bisulfite deamination does not affect the guanine on the complementary strand (see Figure 1). This fact allows in principle to distinguish between sequence variation and bisulfite conversion and therefore to i) avoid wrong inference of the methylation state due to sequence variation and ii) detect sequence variation in the same sample preparation as the methylation levels. Profiling the methylation levels and the genotype of the sample from one experiment will be a very important step towards "putting the DNA back into methylation” ^22^, as the impact and importance of certain DNA sequences on the methylation levels have been recently demonstrated ^23^. To our knowledge, the first program that performed a threshold-based detection of sequence variation in bisulfite sequencing experiments was *NGSmethPipe* ^16^. This program detects sequence variation mainly to avoid wrong inference of the methylation level reporting those genome positions in the output. Only recently, the first state-of-the-art SNP calling algorithm based on the *Genome Analysis Toolkit* ( *GATK*) ^24^ was implemented to detect both methylation levels and sequence variation at high precision in a single experiment ( *Bis-SNP*).

Here we present *MethylExtract*, a multi-threaded tool for methylation profiling and sequence variation detection from alignments in standard BAM/SAM format ^25^. The tool is able to generate high quality methylation maps taking into account SNVs, putative bisulfite failures, reducing also the contribution of sequencing errors by means of the base quality PHRED score ^26, 27^. In addition, it detects sequence variation based on *VarScan* methodology ^28^ reporting all detected SNVs in VCF format ^29^. Therefore, from a single sequencing experiment, *MethylExtract* obtains both the methylation levels and the sequence variation, which will increase the reliability of downstream analyses ^23^. We confirm its usefulness using extensive artificial BS data and a comparison to *Bis-SNP*. We show that while its SNV-calling performance is slightly less specific but more sensitive compared to *Bis-SNP*, *MethylExtract* performs better in methylation profiling, is easier to use and over twice as fast on a typical whole genome experiment.

## Implementation

### Scope and workflow

*MethylExtract* is implemented in Perl and consists of one main script and two auxiliary scripts that are exclusively dedicated to the statistical assessment of the bisulfite error. In general, the program takes standard BAM/SAM file format as input (previously aligned reads) and performs methylation profiling and SNV calling taking into account several error sources like sequencing errors, clonal reads and bisulfite failures. *MethylExtract* writes two output files. First, the methylation information for each cytosine including the coordinates, sequence context (CG, CHG, CHH), number of methylcytosines, read coverage and mean base quality (PHRED) score. The second output file reports the sequence variation in standard VCF format ^29^.

Frequently, whole genome bisulfite experiments include the estimation of the bisulfite conversion rate through a completely un-methylated genome (lambda phage for example). If the bisulfite conversion rate is known, statistical tests can be applied to infer whether an observed methylation level might be only due to failures of bisulfite conversion. The two auxiliary scripts allow i) estimating the bisulfite conversion rate by mapping the bisulfite-treated reads from the un-methylated genome only and ii) to apply a binomial statistics based test to infer the probability that the “real” methylation value lies within a given interval of the observed value.

### Duplicated reads

The PCR step can lead to duplicated (clonal) reads, thus causing a bias in the read coverage. This bias might lead to incorrect inference at positions with allele-specific methylation (genetic imprinting), sequence variation, hemi-methylation, sequencing errors, bisulfite failure or those that are heterogeneous over the cell population. Frequently, the start coordinates of the alignments are used to eliminate duplicates like in *SAMtools* ^25^, adding a criterion to keep the best read among the duplicates. However, those approaches do not take into account that at a heterozygous locus two reads with the same start coordinate could represent two different alleles, thus not being clonal reads. The same applies for loci with genetic imprinting or hemi-methylation. To avoid the elimination of meaningful biological information, *MethylExtract* groups all reads that start at the same position in the genome and that have the same seed nucleotides with Q ≥ ‘minQ’; and selects the read that has the highest number of bases with Q ≥ ‘minQ’ (by default ‘minQ’ = 20) and the longest read in case of equal number of high quality positions. Furthermore, if there are multiple reads with the same selection values, only one will be selected in a random way. Two non-identical reads that align to exactly the same position in the chromosome can represent either the existence of sequence variation or putative clonal reads with a sequencing error in at least one read (disregarding mis-alignments). To restrict the impact of sequencing errors we used only the seed region of the read, i.e. the region with the highest quality. The seed is defined as those nucleotides at the 5´ end of the read (first 26 nt by default) that have a higher PHRED score than ‘minQ’.

Note that the two types of methods, the ones that use only the coordinates and our method using the coordinates and the sequence, have advantages and disadvantages. If the sequence differences are considered, biological meaningful information like sequence variation, genetic imprinting or hemi-methylation is maintained; however, our approach will be vulnerable to sequencing errors and bisulfite errors. The default option is to not perform the detection of duplicated reads, and thus any of the publically available tools can be used optionally to remove clonal reads prior to run *MethylExtract*.

### 5´ end trimming

The first nucleotides can be removed from the 5´ end of the read (3 bp for the *MspI* restriction sites of non-directional representation bisulfite sequencing (RRBS) protocol), as also implemented by *Bismark* ^14^.

### Eliminating reads with putatively high bisulfite conversion failure

The bisulfite conversion error probability of un-methylated cytosines is usually below 1% in modern protocols. However, even for such low values, some positions could be incorrectly profiled, i.e. some methylated cytosines are actually un-methylated. *MethylExtract* implements a method proposed by Lister *et al.* ^30^ to detect those reads with a high number of un-converted cytosines. By default, it eliminates reads with at least 90% of (presumably) unconverted cytosines in non-CpG contexts (Lister *et al.* used ≥ 3 methylated non-CpG cytosines). The default threshold is very conservative and only a rather small fraction of reads will be eliminated. Caution is needed if the user knows that the analyzed species (plants) or tissues (e.g. embryonic stem cells) contain an elevated number of DNA methylation in non-CpG contexts. In those cases, this step should be better skipped as otherwise a bias will be introduced into the analysis.

### Controlling sequencing errors

Sequencing errors are another important cause of incorrect methylation profiling (and SNV calling). The contribution of the individual bases can be controlled by means of the assigned PHRED score (i.e. an upper limit of sequencing error contribution to the wrongly inferred methylation states). For example, when setting PHRED score ≥ 20, thus accepting bases with a probability < 0.01 to be incorrectly called, the contribution of sequencing errors to the overall error would be less than 1%. By default, *MethylExtract* sets the minimum PHRED score to 20 (‘minQ’ parameter) which is then used for both methylation profiling and SNV calling (see below on the determination of the default values).

### SNVs detection

SNVs are the most disregarded error source in the analysis of whole genome bisulfite sequencing data. Most tools would interpret a C to T substitution as an un-methylated cytosine, although a certain number of them are actually SNVs, and therefore this inference would be wrong. A C/T SNV manifests on the complementary DNA strand as an adenine, while bisulfite deamination does not affect the guanine on the complementary strand ^31^ ( Figure 1). The SNVs detection algorithm implemented in *MethylExtract* is an adaptation of the widely used *varScan* algorithm ^28^. The main difference compared to SNV calling from non-bisulfite-treated DNA is the reduced amount of sequence information that can be used to detect sequence variation. The bisulfite treatment converts the un-methylated cytosines into thymines, and therefore, at cytosine positions nucleotides that might result from the bisulfite conversion cannot be used to detect sequence variation. For adenine and thymine, both strands can be used like in re-sequencing experiments. The algorithm works as follows: i) filter out positions that are covered by fewer reads than the minimum read depth (‘minDepthSNV’) – by default ‘minDepthSNV’ is set to 1, thus analyzing all positions that are covered by at least one read; ii) calculate the nucleotide frequencies including all base calls that pass the minimum PHRED score threshold (‘minQ’); iii) discard nucleotides with frequencies below a given threshold (‘varFraction’); iv) calculate a *p-value* for the variant positions (more than two nucleotides above ‘varFraction’) by means of Fisher’s exact test, v) only those positions with a *p-value* below a given threshold are considered as SNVs (‘maxPval’), and vi) the two nucleotides with the highest frequencies are determined as the putative genotype of the sample at this position. Detected sequence variation is reported in VCF output format, which can be used as input for SNP-annotation programs ^32^ or *VCFtools* ^29^.

### Statistical assessment of the bisulfite conversion error

Bisulfite conversion failure has been addressed using binomial statistics for the two possible outcomes; methylated and un-methylated ^33^. However, intermediate biologically meaningful states exist like allele specific methylation (with expected methylation levels of 0.5, if both homologous chromosomes have the same sequencing depth), or the reported partial methylation levels ^30^. Therefore, we developed a statistical test for the methylation levels and not for the methylation state previously proposed ^30, 34^. To apply this test, the user needs to know the bisulfite conversion rate obtained in the experiment. This rate needs to be established using an un-methylated genome (lambda phage, chloroplast, etc). We supply two additional scripts to i) estimate the bisulfite conversion rate using the appropriate experimental data, and ii) associate a *p-value*, based on binomial statistics, to each of the extracted methylation levels, as well as a procedure to control the false discovery rate ^35^.

In order to calculate a *p-value* for a given methylation level, we first need to select an interval as we want to calculate the probability that the real methylation level lies within an interval of the observed methylation level. Once the interval is fixed, we can calculate the number of false methylcytosines that would not change the methylation level, e.g. the methylation level would stay within the error interval.

Once we have detected the maximum number of false methylcytosines that would maintain the methylation level within the error interval, we can calculate the *p-value* by means of the binomial distribution:

$$\text{p}-\mathrm{value}=1-\sum_{k=0}^{fmc}\left(\begin{array}{c} mc\\ k \end{array}\right)p^{k}{\left(1-p\right)}^{mc-k}$$

being: *p* the bisulfite error rate, *mc* the number of observed methylcytosines at a given position and *fmc* the maximum number of allowed false methylcytosines. The *p-value* corresponds then to the probability to find more than *fmc* false methylcytosines at this position, e.g. the probability that the real methylation level lies outside the defined error interval.

To illustrate the method, let’s assume that we have a position that is covered by 21 reads with 17 methylcytosines. In this situation, we would have a methylation level of 0.81. If we fix the error interval at 0.1, we could accept up to 2 false methylcytosines. For two false methylcytosines, the methylation level would be (17–2)/21 = 0.714 which lies within the error interval of 0.81–0.1 < 0.714 while 3 false methylcytosines would lead to a methylation level of 0.67 which lies outside the tolerated error interval. Note that the coverage depth of the position (number of reads) does not appear in the equation, but it does to calculate the maximum number of false methylcytosines. In this way, a higher coverage will lead to a higher number of allowed false methylcytosines and therefore to smaller *p-values*. Finally, we implemented the Benjamini–Hochberg step-up procedure ^35^ to control for the false discovery rate in multiple testing. This step can be optionally activated by the user.

## Results

### General comparison to other available tools

*MethylExtract* is currently one of the programs with most implemented features related to quality control. Together with *Bis-SNP* it is the only program that detects sequence variation, both to avoid incorrect methylation profiling and to assess the genotype of the used sample. Table 1 shows a comparison of the main features of all programs that allow methylation profiling from aligned reads. Apart from the used method to call the sequence variation, another important difference between *MethylExtract* and *Bis-SNP* is the number of scripts involved to run a full analysis. *Bis-SNP* requires the execution of: i) 3 scripts to sort, add read group tags (required by *GATK* tools) and mark duplicates, ii) 4 scripts to realign the reads and recalibrate the base quality score, iii) 2 scripts to obtain and sort the SNVs and number of methylcytosines and iv) an additional script to calculate the methylation levels on a standard format. In summary, *Bis-SNP* needs 10 different scripts to process reads from bisulfite-treated experiments. On the other hand, *MethylExtract* unifies all analysis steps into a single program which makes it especially suitable for users without a bioinformatics background. Another feature that is currently unique to *MethylExtract* is the possibility to assess the bisulfite failure in a statistical way. In order to achieve this, *MethylExtract* provides an auxiliary script to estimate the bisulfite conversion rate, and a second script that calculates the probability that the observed methylation level lies outside the selected interval of the real methylation level due to bisulfite conversion failures.

**Table 1.** Comparison of *MethylExtract* with different programs for methylation profiling and SNV calling.

FEATURES ^#^/SOFTWARE	MethylCoder	BS_SEEKER	BRAT-BW	BSMAP/RRBSMAP	Bismark	Bis-SNP	MethylExtract
**Input formats**	*	*	*	Sam	Sam	Bam	Sam/Bam
**5´ Trim**	No	No	Yes	No	Yes	Yes	Yes
**Bisulfite failure**	No	No	No	No	No	No	Yes
**Minimum depth**	No	No	No	Yes	No	No	Yes
**Base call errors**	No	No	No	No	No	Yes	Yes
**SNVs calling**	No	No	No	No	No	Yes	Yes
**Methylation output** **formats**	*	*	*	*	*, bed	vcf, bed, wig	*, bed, wig
**Variation output** **formats**	-	-	-	-	-	vcf	vcf

^#^ Input formats: input formats used by each software. 5´ trim: allows the trimming of the 5´ end of the reads. Bisulfite failure: implementation of a step to discard reads where the bisulfite might have failed converting the un-methylated cytosines. Minimum depth: allows the user to discard positions with low coverage. Base call errors: discards positions that do not exceed a given minimum PHRED score value. SNVs calling: detects variation that can lead to wrong methylation levels or context estimation. Methylation output formats: available formats for the methylation results. Variation output formats: output formats for the sequence variation results. The asterisk (*) represents a non-standard input or output format, or the impossibility of extracting the methylation ratios from other alignment tools. The dash (-) represents the inexistence of SNV output format, because the software does not allow to detect them.

### Impact of SNVs on methylation levels

**Figure 2.**
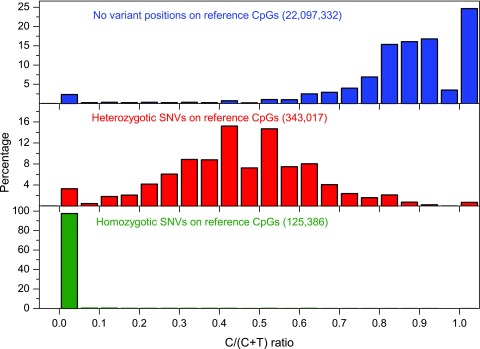
Distribution of C/(C+T) ratios for cytosines within the CpG context in the H1 cell line. C/(C+T) values for cytosines at non-variant and variant (homo- and heterozygotic) positions were shown. The minimum read coverage was set to 10 reads.

As mentioned above, sequence variants can lead to incorrect inference of methylation values. Figure 2 illustrates the impact of C/T variation on the methylation values (C/(C+T) ratio) within CpGs contexts. Around 470,000 SNVs within CpG contexts (affecting to 2.08% of the CpG contexts on the genome) covered by at least 10 reads have been detected by *MethylExtract* in Lister’s H1 dataset ^30^. Figure 2 shows the methylation levels for non-variant positions (both alleles coincide with the reference) and for the variant sites, both in homozygosis and heterozygosis. The observed distribution of the methylation levels without variation has two maxima close to 0 and 1, which is similar to previous studies ^30^. However, for heterozygous positions detected by *MethylExtract*, the methylation levels present a local maximum at approximately 0.5 (the T allele on one of the parental chromosomes biases the methylation levels to intermediate values, if the C allele is methylated) and a peak at 0 (if the C allele is un-methylated). Finally, for the homozygous positions where both chromosomes present the T allele, most of the methylation values are exactly 0. However, we know that no cytosine exist at those locus in the analyzed sample and therefore these values are incorrect and should be eliminated from the analysis. The incorrectly inferred methylation values for variant positions, both in homozygosis and heterozygosis, stress the need to detect and remove them from the analysis. For example, a CpG position with C→T SNV on both homologous chromosomes is eliminated by *MethylExtract,* as actually at this position no CpG exists in the sample. Furthermore, *MethylExtract* outputs the detected genotype of all profiled positions and therefore heterozygotic loci can be detected easily by the user and treated apart if wished.

### Methylation profiling and SNV calling quality

*MethylExtract* implements several quality controls and is among the programs with most implemented features. Main features of *MethylExtract* are compared in Table 1 to a number of other, widely used programs. The implementation was validated in several ways. *MethylExtract* takes aligned reads as input and therefore we first compared the methylation profiling quality achieved on artificial bisulfite data when using two different tools for aligning bisulfite-treated reads; *NGSmethPipe* ^16^ and *Bismark* ^36^. Next, we quantify the correctly profiled methylation levels and SNVs as a function of the main quality parameters using *NGSmethPipe* as aligner. Finally the predictive power of *MethylExtract* to detect methylation levels and sequence variation was compared to *Bis-SNP* ^24^, both in terms of sensitivity and positive predictive value as it was proposed for datasets for which the number of true negatives tend to be much higher than false positives ^37^.

***Generation of artificial BS data.*** For all further comparisons we will use artificial bisulfite data. The usage of this kind of data for benchmarking has the advantage that the true methylation levels and genotypes are known for each position, which is not true when using other experimental methods like microarrays as a golden standard. Artificial sequencing data has been used before in other studies assessing the SNV prediction quality of different algorithms ^38^. To generate the artificial bisulfite data we used *DNemulator* ^18^. We obtained two datasets from the human contig GL000022.1 (11.2Mb), one with all CpGs completely methylated, and the other one with all CpGs completely un-methylated. *DNemulator* allows also simulating the genotypes of a diploid genome by introducing the sequence variation from a set of confirmed SNPs (dbSNP135) ^39^. Finally, we simulate a bisulfite conversion rate of 99%. The read quality scores are taken from real experimental data (Lister’s H1 dataset ^30^). All together, we generated artificial bisulfite sequencing datasets at two different coverages; 15× and 20× which corresponds to the coverage usually achieved in whole genome bisulfite sequencing experiments.

***MethylExtract with NGSmethPipe and Bismark input.*** *NGSmethPipe* ^16^ is a tool to align bisulfite-treated reads which was developed by our group. It is based on the *Bowtie* aligner and uses a 3-letter alphabet to map the bisulfite-treated reads. The program implements a pre-processing to improve the mapping accuracy ^18^ and an alignment seed extension in order to increase the number of mapped reads.

**Figure 3.**
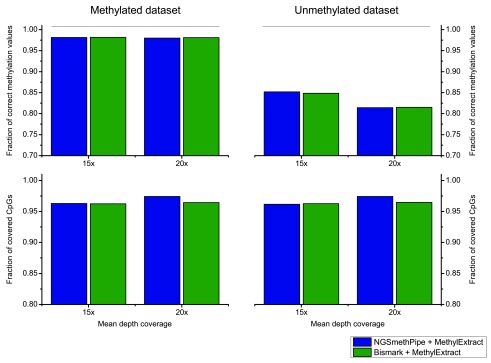
CpGs methylation profiling comparison for alignment methods. The results obtained from *MethylExtract* (correctly profiled methylation values and CpG coverage) using two bisulfite short read aligners, *NGSmethPipe* and *Bismark* are compared. The results are nearly independent of the used alignment algorithm.

We launched both, *NGSmethPipe* and the well-established *Bismark* tool with default options to obtain the SAM/BAM input. Next we used *MethylExtract* on both input files to obtain the number of covered CpGs and the number of correctly recovered methylation values. Note that we know the correct methylation value for each position due to the use of artificial bisulfite data. A position is considered as correctly profiled, only if the obtained methylation value is identical to the real value. Figure 3 shows the result of this comparison. It shows that the obtained CpG coverage and number of correctly profiled positions is nearly identical both as a function of read coverage (15× and 20×) and for the methylated and un-methylated input data. The only remarkable difference is that *NGSmethPipe* leads to a slightly higher CpG coverage at 20× for both data sets. Nevertheless, the main conclusion is that *MethylExtract* yields nearly identical results for input sets obtained from *NGSmethPipe* and *Bismark*.

**Figure 4.**
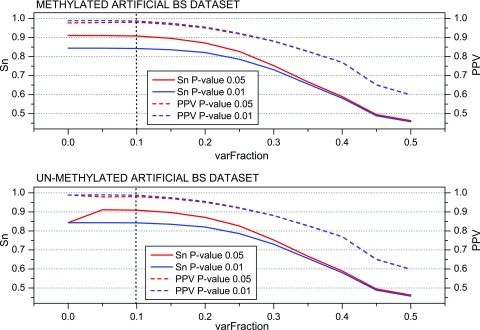
*MethylExtract* SNV calling as a function of the minimum relative nucleotide frequency (‘varFraction’). The figures show the sensitivity (Sn) and the positive predictive value (PPV) for SNV detection using two different p-value thresholds. The graphs are based on the methylated (top) and un-methylated (bottom) artificial bisulfite datasets at a mean 20× read coverage.

***Analysis of the MethylExtract quality parameters.*** Next, we aimed to assess the impact of certain quality parameters implemented in *MethylExtract* on the methylation profiling and SNV calling capacity. To detect sequence variation, *MethylExtract* relies on two main parameters, i) the relative nucleotide frequencies (‘varFraction’) and ii) the corresponding *p-value*. The ‘varFraction’ parameter determines if a position shows putatively variation: the position is analyzed only if at least one nucleotide that differs from the reference sequence has relative frequencies higher than ‘varFraction’. Only for these positions the corresponding *p-value* is calculated by means of a Fisher exact test. Figure 4 shows the impact of these parameters on the prediction sensitivity (Sn) and positive predictive value (PPV). Sequence variation is best detected by setting the ‘varFraction’ threshold close to 0.1 (yielding around 91% Sn and only 2% of false positives at a statistical significance of 0.05). If the ‘varFraction’ threshold is increased further, the probability to eliminate heterozygous loci increases steadily for positions with high bias in the read coverage between the two homologous chromosomes. If the *p-value* threshold is set to 0.01, a small increase in positive predictive value (PPV) is observed, but it causes a strong decrease in sensitivity. Therefore, we determined a ‘varFraction’ of 0.1 and a *p-value* threshold of 0.05 as the best (default) parameters to detect sequence variations.

**Figure 5.**
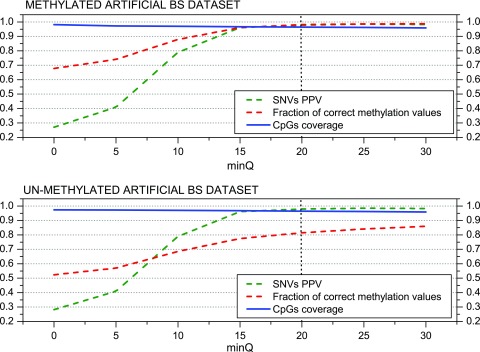
*MethylExtract* SNV calling and methylation profiling as a function of the base quality. Both graphs show the positive predictive value (PPV) for SNV calling and the fraction of correctly profiled CpG methylation values (methylation profiling) as a function of the minimum base quality (PHRED score parameter ‘minQ’). The graphs are based on the methylated (top) and un-methylated (bottom) artificial bisulfite datasets at a mean 20× read coverage. Y-axis represents SNV PPV, Fraction of correct methylation values and CpG coverage. All of them vary between 0 to 1 therefore being represented together.

The minimum base quality (‘minQ’) and the coverage depth (‘minDepthMeth’ for the methylation profiling) thresholds might be also important parameters to control de quality of methylation profiling and SNV calling. To analyze the impact of the minimum PHRED score parameter (‘minQ’) we fix the minimum read coverage (‘minDepthMeth’) in 3, as suggested by Laurent *et al.* ^40^, ‘varFraction’ = 0.1 and ‘maxPval’ = 0.05 (default values derived above). Figure 5 shows the fraction of correctly profiled methylation values and the PPV for SNVs. It can be seen that the correctly profiled positions increase approximately 31% (from 68% to 99%) and the SNVs around 71% (27% – 98%), when the minimum PHRED score is increased from 0 (all base calls are accepted) to 30 (0.001 error probability). The major difference between the methylated and un-methylated datasets is observed for the profiling of the methylation level for which the percentage increases only from approximately 52% to 86%. The simulated bisulfite conversion failures will affect mainly un-methylated positions which can explain the observed differences. These results confirm that the ‘minQ’ threshold is critical to obtain high quality methylation profiling and genotyping results. The default value was set to 20 as higher values will lead to a coverage reduction compromising the SNV calling sensitivity.

### Comparison with *Bis-SNP*

The comparison between *MethylExtract* and *Bis-SNP* needs to be based on identical alignment input files in BAM/SAM format. We obtained these files in a two-step process: First, we trim the input reads as it was done by Lister *et al.* ^30^ and second, we align the bisulfite treated reads to the reference genome using *Bismark* ^36^ with default parameters. Note that we based this comparison on *Bismark*, as the realignment and recalibration steps implemented in *Bis-SNP* require the read mapping quality, which is currently not available in *NGSmethPipe*.

**Figure 6.**
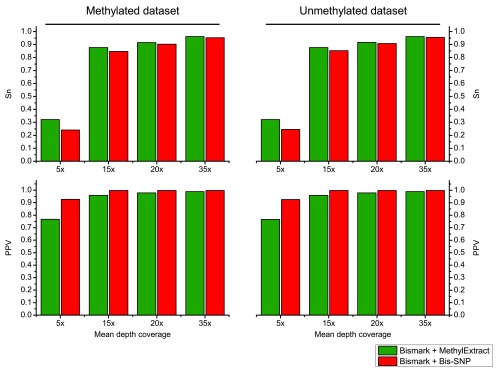
Comparison of SNV calling between *MethylExtract* and *Bis-SNP*. The top graph shows the sensitivity (Sn) and the bottom graph the specificity (PPV) obtained for the methylated and un-methylated artificial bisulfite datasets at two different mean coverages (5×, 15×, 20× and 35×).

**Figure 7.**
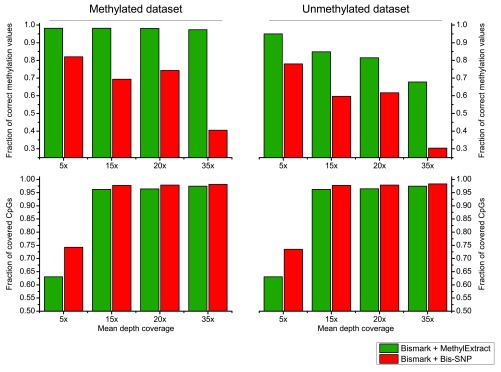
Comparison of CpG methylation values between *MethylExtract* and *Bis-SNP*. Both methods are compared in terms of fraction of correctly profiled CpG methylation values (top) and the fraction of recovered CpG positions (bottom).

**Supplementary Figure 1.**
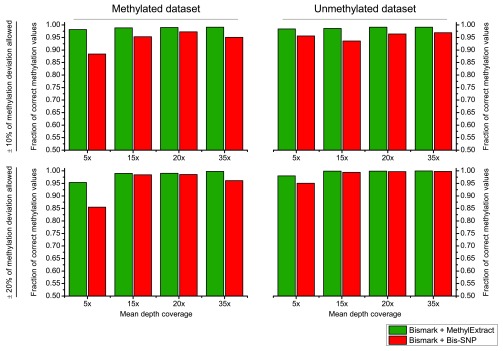
Methylation profiling comparison *MethylExtract* and *Bis-SNP* using relaxed criterion. Both methods are compared in terms of fraction of correctly profiled CpG methylation values. The upper part of the graph shows the result allowing up to 10% deviation from the real methylation values, while the lower part shows the outcome increasing this range to 20%. The analyses were done for unmethylated and methylated datasets at four different coverages (5×, 15×, 20× and 35×).

Both methods were used with default parameters. We first compared the detection of sequence variation (SNVs) in terms of Sn and PPV. Figure 6, shows that in general *Bis-SNP* is more specific (between 1.9% and 3.9% higher PPV), being *MethylExtract* more sensitive (between 1% and 3.1% higher Sn). This trend can be seen for both artificial bisulfite datasets as well as for both read coverages. However, when comparing the fraction of correctly recovered methylation values, drastic differences can be seen ( Figure 7). Furthermore, when the criteria for correctly profiled methylation values are relaxed, *MethylExtract* still yields higher fractions than *Bis-SNP* ( Supplementary Figure 1). While *Bis-SNP* yields a slightly higher number of covered positions (Fraction of covered CpGs), *MethylExtract* is more specific. In all four comparisons using the stringent criteria (no deviation from the real methylation values is allowed), *MethylExtract* yields over 20% more correctly profiled positions compared to Bis-SNP. One explanation for this difference might be the PHRED score quality threshold implemented in *MethylExtract*.

### Runtime comparison to *Bis-SNP*

As mentioned before, only *Bis-SNP* and *MethylExtract* perform the detection of SNVs which constitutes an additional CPU demanding task. Therefore, we only compared these two programs in terms of CPU time using a reduced Lister’s H1 dataset ^30^ on a 24 core Intel(R) Xeon(R) CPU X5650 2.67GHz machine. Available memory is crucial for both methods. In order to not bias the comparison, we limited the available memory to 15GB for both programs allowing up to 15 threads. Both programs were tested using a 11GB BAM input file. After aligning with *Bismark*, we carried out the entire process for both tools (from the aligned reads to the methylation and SNV profiling). *MethylExtract* needed 6 hours 2 minutes to process the entire dataset including the sorting by coordinates and the removal of duplicated reads. *Bis-SNP* spend 2 hours 47 minutes sorting the file and removing putative clonal reads, 9 hours and 36 minutes realigning and recalibrating the reads, and 15 hours 54 minutes genotyping and retrieving the methylation levels. Therefore, it seems that *MethylExtract* is notably faster than *Bis-SNP* (approximately 4.5 times on this whole genome data set).

## Conclusions

We present a user-friendly tool for methylation profiling and SNV calling in whole genome bisulfite sequencing experiments. *MethylExtract* takes standardized input formats (BAM/SAM) and writes out likewise broadly used file formats like WIG, BED and VCF. To show its usefulness, we compared it to *Bis-SNP*, a recently published method that is very similar in scope. Although *Bis-SNP* is more specific (less false positive predictions) in the detection of SNVs, *MethylExtract* is more sensitive (higher number of recovered SNVs). However, the main advantages of *MethylExtract* when compared to *Bis-SNP* seem to rely in the higher percentage of correctly profiled methylation values, as it reaches values over 20% higher compared to *Bis-SNP*. Other aspects that favor *MethylExtract* are its user-friendliness (everything is implemented into one script) and the run-time in comparison to *Bis-SNP* (over 4 times faster in a whole genome bisulfite sequencing experiment).

## Availability and requirements

*MethylExtract* is freely available. The source code, the tutorial and artificial bisulfite datasets can be downloaded from the page http://bioinfo2.ugr.es/MethylExtract/ and are also permanently accessible from 10.5281/zenodo.8351 ^42^.

## List of abbreviations used

5meC: DNA methylation at cytosine carbon 5 position; SNV; Single Nucleotide Variation; WGBS: whole genome bisulfite sequencing; SNP: Single Nucleotide Polymorphism; PPV: positive predictive value; PHRED score: the quality score to each base call assigned by the program PHRED; SAM format: Sequence Alignment/Map format used for storing large nucleotide sequence alignments; BAM format: the compressed binary version of the SAM format.
