# Supplementary material for: MethylExtract: High-Quality methylation maps and SNV calling from whole genome bisulfite sequencing data
Source: F1000Res. 2014 Feb 21;2:217. Originally published 2013 Oct 15. [Version 2] doi: 10.12688/f1000research.2-217.v2 (PMC3938178; doi:10.12688/f1000research.2-217.v2)
Supplement: Peer review history

# Peer review: Referee response for version 2

**DOI:** 10.5256/f1000research.3889.r3824

**Published:** 2014-05-22

**Authors:** Michael Stadler

**Affiliations:** 1 Friedrich-Miescher Institute for Biomedical Research, Basel, Switzerland

The incompatibility with older Perl versions has been fixed, and the script (v1.7) runs now on RHEL6 (Perl v5.10.1).

I agree that a ROC analysis for the results presented in Fig. 6 would require subsampling of C's to achieve a better balance between methylated and non-methylated states. It is unfortunate that the authors choose not to do that, because it limits the value of this analysis, however it should not preclude indexing of the article.

I have read this submission. I believe that I have an appropriate level of expertise to confirm that it is of an acceptable scientific standard.

# Peer review: Referee response for version 2

**DOI:** 10.5256/f1000research.3889.r3823

**Published:** 2014-04-01

**Authors:** Felix Krueger

**Affiliations:** 1 Babraham Bioinformatics, Babraham Institute, Babraham, UK

All my comments were already satisfactorily, or will be addressed in an upcoming version of MethylExtract.

I have read this submission. I believe that I have an appropriate level of expertise to confirm that it is of an acceptable scientific standard.

# Peer review: Referee response for version 1

**DOI:** 10.5256/f1000research.2616.r3164

**Published:** 2014-01-27

**Authors:** Felix Krueger

**Affiliations:** 1 Babraham Bioinformatics, Babraham Institute, Babraham, UK

**General comments**

MethylExtract is a new tool that implements several QC related steps on already aligned Bisulfite-Seq data. This includes the deduplication of clonal reads, filtering based on base call quality, identification of potential bisulfite conversion errors and most notably the detection of single nucleotide variants (SNVs) that would affect methylation calls, the latter of which is until now only being performed by Bis-SNP.

After reading the documentation it was straight forward to set MethylExtract off, and it ran to completion in an acceptable time frame (~1d 7h for one lane of HiSeq data aligned against the human genome using Bismark). I liked that the deduplication tool that would potentially allow reads aligning to the same position in the genome to pass if they contain SNVs and thus originate from two distinct alleles (even though it is probably questionable whether one would expect to see this sort of reads a lot for large eukaryotic genomes at moderate read depth such as 15x).

Even though I've got a few questions about how certain things are handled or documented in the current implementation of MethylExtract, I was quite impressed at how easy it was to get hold of SNV information using just one command. I am sure MethylExtract will prove a useful tool in the genuine analysis of bisulfite data.

**Handling paired-end alignments**

The only time I found mention of paired-end reads was in the explanation of the deduplication option 'delDup' in the MethylExtract Manual. One can further see that the options 'tagW' and 'tagC' for FLAG values of reads aligning to the Watson and Crick strands default to 0 and 16, respectively, which are standard FLAG values for forward or reverse mapping single-end reads. If MethylExtract currently only handles single-end experiments this is fair enough, even though it would be a serious limitation seeing that most data we generate is in fact paired-end data. Paired-end reads will have at least four different FLAG values, so it is unclear if and how one would specify these? Would the SNV detection would still work with paired-end reads? Are there options to remove overlapping parts in the reads? In any case, I think paired-end reads should be documented better, both in the manuscript and the manual.

**Handling SNV positions**

If I understood it correctly, CpG positions with a homozygous C to T SNV are eliminated by MethylExtract before reaching the final methylation output, but does this also happen for heterozygous loci? The manual mentions: "(v) SNV can be detected and removed" - can they or are they?

It seems that MethylExtract reports only a single position (presumably the most 5' one?) for cytosines in CG or CHG context (this could also be mentioned in the manual). Are symmetrical cytosine positions completely eliminated from the output if SNVs are detected on at least one strand? Similarly, is the context of a cytosine determined purely by the genomic sequence or would a homozygous SNV effectively also change a C's context (e.g. would an A to G SNV from 'CAG' to 'CGG' change the context from CHG to CG?). I would find it useful to read some more information on how these cases are dealt with, maybe in the User Guide? Finally, to make it a bit more user-friendly I would welcome an option to include or exclude positions from the output specifically that were detected as homozygous or heterozygous SNVs.

**M-bias**

MethylExtract addresses several important aspects affecting the accuracy of BS-Seq experiments, however it doesn't mention the issue of methylation bias (M-bias) in the reads (described in Hansen *et al.,* 2012). M-bias may have several causes, such as 5' bisulfite conversion failure (described in BSeQC - Lin *et al., *2013), fill-in bias during library construction which is especially pronounced for paired-end reads (as an example see read 2 in this report: http://www.bioinformatics.babraham.ac.uk/projects/bismark/PE_report.html) or other technical reasons (e.g. PBAT libraries, an example report is available here: http://www.bioinformatics.babraham.ac.uk/projects/bismark/SE_report.html). Are there any plans to take M-bias into consideration in future versions of MethylExtract?

**5' end trimming**

Both the manuscript and the MethylExtract manual mention that there is an option to ignore bases at the 5' end of reads, e.g. "3 bp for the *MspI* restriction sites of reduced representation bisulfite sequencing (RRBS) protocol". While the option is certainly useful, e.g. for the removal of M-bias in the reads, it should be noted that for standard (= directional) RRBS libraries the first 3bp should reflect the true genomic methylation state of the *MspI* site and do not have to be ignored. The situation is somewhat different for non-directional reads or when reading through filled-in positions at the 3' end of reads. We have tried to illustrate this in a bit more detail in this brief RRBS guide ( http://www.bioinformatics.babraham.ac.uk/projects/bismark/RRBS_Guide.pdf).

**Very minor**

The version of MethylExtract hosted on zenodo.org which I downloaded first was outdated (v1.3) and failed to run at some point. The latest version from http://bioinfo2.ugr.es/MethylExtract/ (v1.4) worked fine.

I have read this submission. I believe that I have an appropriate level of expertise to confirm that it is of an acceptable scientific standard.

# Peer review: Referee response for version 1

**DOI:** 10.5256/f1000research.2616.r2607

**Published:** 2014-01-13

**Authors:** Jörn Walter

**Affiliations:** 1 Institute of Genetics/Epigenetics, University of Saarland, Saarbrücken, Germany

The mapping and calling of cytosine methylation in whole genome bisulfite sequencing is a challenging task. Following sequence alignment, the localisation and scoring of reliable and quantitative positional methylation information requires a number of control functions including the detection and high quality scoring of SNVs. So far BiSNP has been used as the major tool for these tasks. The MethylExtract tool now offers a (slightly) improveed software suite compiling state of the art (BiSNP-like) features with additional QCs. One advantage of MethylExtract is that the package can be executed in a single PERL script. Compared to BiSNP, MethylExtract reduces the error rate of false SNV calling by including an optimized PHRED score and  controlling for bisulfite conversion error rates. MethylExtract accepts SAM and BAM alignment files and cretaes an independent SNV output file (VCF). With SAM as an input MethylExtract runs about 2x faster compared to BiSNP. A performance test on 15x and 20x  artificial test alignment sets shows a better performance of MethylExtract in comparison to BiSNP but only with respect to specificity, while BiSNP has the better sensitivity. Unfortunately the authors did not run a direct comparison on real datasets. Overall MethylExtract is a nice compilation of surely useful tools for a comprehensive and quality controlled WGBS data analysis. The key features of the package are nicely documented. I only have my doubts that conversion rate errors calculated on spiked in control DNA really generates a meaningful background correction.

I have read this submission. I believe that I have an appropriate level of expertise to confirm that it is of an acceptable scientific standard.

# Peer review: Referee response for version 1

**DOI:** 10.5256/f1000research.2616.r2103

**Published:** 2013-10-25

**Authors:** Michael Stadler

**Affiliations:** 1 Friedrich-Miescher Institute for Biomedical Research, Basel, Switzerland

The authors present the tool MethylExtract that combines extraction of methylation states and SNV calling, given alignments of bisulfite-converted reads to a reference in SAM/BAM format. Methylated CpG are mutation hotspots - dealing with SNVs is therefore an important part of any analysis of Bis-seq data.

The main functions of MethylExtract are implemented in a single Perl script, which should make it easy to use - unfortunately I could not verify this because it failed to run in my environment (see below).

The performance of MethylExtract is evaluated using simulated sequence data (completely methylated or completely unmethylated containing sequencing errors and SNVs) and compared to Bis-SNP, a conceptually similar tool that is based on the GATK variant calling package. The simulations cover most important aspects of the tool; however, the paper would benefit from extended simulations and a test on a real dataset. For example, the current evaluation does not cover Bis-seq datasets with very low or very high coverage or intermediate methylation levels (see minor issues below).

The paper is clearly written, and the conclusions are supported by the presented results.

**Major issues:**

- The **MethylExtract perl script** (version 1.3) did not run in my environment (RHEL 6, with Kernel 2.6.32-220.7.1.el6.x86_64, perl 5.10.1 built for x86_64-linux-thread-multi). Trying to run the main script resulted in a compilation error:
    >perl MethylExtract_1.3.pl
    Type of arg 1 to keys must be hash (not hash element) at MethylExtract_1.3.pl line 318, near "}) "
    Execution of MethylExtract_1.3.pl aborted due to compilation errors.
  It is possible that the problem lies in the combination of the script and the test environment. However, the test environment fulfills the stated requirements and dependencies.
- **Figure 6** and corresponding text: A single point comparison of Sn/PPV between MethylExtract and Bis-SNP, both with default parameters, is not very informative. Typically there is a trade-off between sensitivity and specificity which can be influenced by the choice of parameter values such as the score or P value cutoffs. It is possible that with slightly altered parameter values, the improved Sn/reduced PPV of MethylExtract compared to Bis-SNP turn into the opposite. The two tools should therefore be compared using varying parameter settings or cutoffs (altering the trade-off between Sn and PPV) and then relating the resulting specificity and sensitivity in an ROC analysis. The same applies in principle to the results presented in Figure 3.

**Minor issues:**

- **Simulations - readout:** The current evaluation of "correct methylation" requires exact identity of simulated and estimated methylation states. This criterium is very stringent yet may not be able to uncover systematic problems. In practice, a very small deviation from the true methylation level may be tolerable. For illustration: A tool that produces many incorrect values that are off only by a small amount may be preferable to a tool that produces fewer incorrect values that are several-fold off. I would suggest using a continuous measure of performance (e.g. the differences between true and estimated methylation levels) or to allow for a minimum deviation.
- **Simulations - methylation levels:** In the introduction, the authors point out the value of methylation levels as opposed to methylation states. Also, intermediate methylation is present in virtually all real world Bis-seq data sets. The simulations should take this into account and also contain C's with intermediate methylation levels (e.g. around 50% methylation).
- **Simulations - coverage:** The current simulations are performed at 15- and 20 fold coverage and the two yield very similar results. More informative differences in performance may be observed when simulating data at even lower (~5-fold) or higher (>30-fold) coverage, which are commonly found in published Bis-seq datasets.
- **Runtime comparison:** It's surprising that even though MethylExtract supports BAM input, SAM.gz was used for runtime measurements, while Bis-SNP was reading from BAM input. Unpacking of alignments from BAM files is CPU-intensive, and I wonder if MethylExtract would take more time if it was run on the same input as Bis-SNP.
- **Table 1:** MethylExtract is listed to support both SAM and BAM inputs. However, it does not directly read BAM files, but converts them to SAM using SAMtools. Using such a conversion, BSMAP and Bismark also support BAM input. I would suggest not to discriminate between SAM and BAM inputs in the table to avoid confusion based on this subtle difference.
- **Impact of parameter choice when analyzing real world data:** For some parameters (e.g. "duplicated reads filter" and "elimination of bisulfite conversion failure"), it is unclear how they would impact results in a real world analysis. A comparison of the results obtained on an experimental dataset with different parameter values could identify sensitive parameters and guide users when choosing parameters for their own analysis.
- **Figure 5:** The labels of the two y-axes are missing. In addition, CpG coverage (blue line) was probably scaled to be plotted on the same axis; if that is the case, it should be described in the legend and/or indicated in the plot.

I have read this submission. I believe that I have an appropriate level of expertise to confirm that it is of an acceptable scientific standard, however I have significant reservations, as outlined above.
